# Availability of genome-matched therapy based on clinical practice

**DOI:** 10.1007/s10147-024-02533-z

**Published:** 2024-04-26

**Authors:** Naomi Hayashi, Seiichi Mori, Akihiro Ohmoto, Ippei Fukada, Masumi Yamazaki, Mari Hosonaga, Xiaofei Wang, Arisa Ueki, Kazuma Kiyotani, Akiko Tonooka, Kengo Takeuchi, Shunji Takahashi

**Affiliations:** 1 Department of Genomic Medicine, Cancer Institute Hospital of Japanese Foundation for Cancer Research, 3-8-31 Ariake, Koto, Tokyo 135-8550 Japan; 2 Department of Clinical Genetic Oncology, Cancer Institute Hospital of Japanese Foundation for Cancer Research, 3-8-31 Ariake, Koto, Tokyo 135-8550 Japan; 3 Project for Development of Innovative Research On Cancer Therapeutics, Cancer Precision Medicine Center, Japanese Foundation for Cancer Research, 3-8-31 Ariake, Koto, Tokyo 135-8550 Japan; 4 Department of Medical Oncology, Cancer Institute Hospital of Japanese Foundation for Cancer Research, 3-8-31 Ariake, Koto, Tokyo 135-8550 Japan; 5 Memorial Sloan Kettering Cancer Center, Human Oncology and Pathogenesis Program, 1275 York Avenue, New York, NY 10065 USA; 6 Department of Breast Medical Oncology, Cancer Institute Hospital of Japanese Foundation for Cancer Research, 3-8-31 Ariake, Koto, Tokyo 135-8550 Japan; 7 Center for Advanced Medical Development, Cancer Institute Hospital of Japanese Foundation for Cancer Research, 3-8-31 Ariake, Koto, Tokyo 135-8550 Japan; 8 Project for Immunogenomics, Cancer Precision Medicine Center, Japanese Foundation for Cancer Research, 3-8-31 Ariake, Koto, Tokyo 135-8550 Japan; 9 Laboratory of Immunogenomics, Center for Intractable Diseases and ImmunoGenomics (CiDIG), Health and Nutrition (NIBIOHN), National Institutes of Biomedical Innovation, 7-6-8, Saito-Asagi, Ibaraki City, Osaka 567-0085 Japan; 10 Division of Pathology, Cancer Institute, Japanese Foundation for Cancer Research, 3-8-31 Ariake, Koto, Tokyo 135-8550 Japan; 11 Department of Pathology, Cancer Institute Hospital, Japanese Foundation for Cancer Research, 3-8-31 Ariake, Koto, Tokyo 135-8550 Japan; 12 Pathology Project for Molecular Targets, Cancer Institute, Japanese Foundation for Cancer Research, 3-8-31 Ariake, Koto, Tokyo 135-8550 Japan

**Keywords:** Comprehensive genomic profiling, Genome-matched therapy availability, Practical strategy

## Abstract

**Background:**

Comprehensive genomic profiling (CGP) provides new opportunities for patients with advanced cancer to receive genome-matched therapies, but the availability rate of these remains low. We reviewed our CGP cases and suggested possible strategies to improve the current status from a clinical perspective.

**Methods:**

Druggable genomic alterations and barriers to accessing genome-matched therapies were investigated in 653 patients with 30 various types of cancers who underwent CGP.

**Results:**

While the availability rate of genome-matched therapies as a whole was 9.5%, CGP was useful in some cancer types. Patients with thyroid cancer and lung cancer harbored druggable genomic alterations at high rates, while sarcoma rarely harbored these alterations (100%, 76%, and 15.2%, respectively). In contrast, the availability rate of genome-matched therapies was highest in patients with sarcoma and head and neck cancer (HNC) (60% and 40%, respectively). One hundred thirteen patients (63.5%) had multiple barriers to accessing genome-matched therapy. Of 178 patients, 21 patients (11.8%) could not be considered for genome-matched therapies solely because of the deterioration of their performance status.

**Conclusion:**

This study demonstrated the usefulness of CGP for patients with sarcoma and HNC in addition to lung cancer in clinical practice. Performing CGP at the front line has the potential to improve the availability of genome-matched therapy.

**Supplementary Information:**

The online version contains supplementary material available at 10.1007/s10147-024-02533-z.

## Introduction

Next-generation sequencing (NGS) is a DNA-sequencing technology that performs sequencing from millions of small fragments of DNA in parallel [1]. NGS-based comprehensive genomic profiling (CGP) enables the detection of large numbers of cancer-associated genomic alterations at once. Results from CGP can guide the use of genome-matched therapies such as molecular targeted drugs or immune checkpoint inhibitors and can lead to clinical trials that provide new opportunities to access agents other than approved drugs. Several studies reported from western countries have evaluated the usefulness of CGP and have reported the availability rates of 10–20% for genome-matched therapies [2–4]. The availability rate of those drugs remains low even present, after several years. Recently, several experts suggested performing CGP on the front line to recruit patients in the best condition and at the best timing possible [5, 6]. However, barriers to accessing genome-matched therapies are not only related to the patient's condition. Hilal et al. reported that the most common barrier was ongoing standard treatment or no available therapeutic option, while deterioration of PS was less common [2]. Although performing CGP on the front line has the potential to improve the availability rates of genome-matched therapies, it is uncertain what proportion of patients can benefit from this approach.

The European Society for Medical Oncology (ESMO) guidelines recommend the routine use of CGP for four types of cancer: non-squamous cell lung cancer, prostate cancer, ovarian cancer, and bile duct cancer [7]. Patients with these cancer types have a high probability of harboring druggable genomic alterations, and a single large panel test is considered preferential to multiple companion diagnostic devices (CDx) in terms of cost and turnaround time (TAT). However, in clinical practice, CGP is often performed after CDx, and there may be differences between the guidelines and clinical practice regarding recommended cancer types. Ida et al. reported that CGP was useful for common cancers, while Kondo reported that it was useful for rare cancers [6, 8].Thus, clinical usefulness of CGP remains unclear. Hence, we conducted a large clinical study to assess the clinical usefulness of CGP. The availability of genome-matched therapies across cancer types as well as the barriers to accessing these drugs were investigated. The aim of this study was to assess a clinical usefulness of CGP and suggest possible strategies to improve the availability of genome-matched therapies.

## Patients and methods

### Patients

We retrospectively analyzed collected data from 683 consecutive patients who underwent CGP covered by Japanese insurance policies in our department between December 2019 and July 2022. Thirty patients were excluded because the CGP results were not available due to low specimen quality. Patient data comprised age, sex, Eastern Cooperative Oncology Group Performance Status (ECOG PS) [9], primary organ, histology, and genomic alterations. Data-cut off was March 2023.

### CGP and the definition of druggable genomic alterations

Two tissue-based CGP assays and one liquid-based CGP assay are covered by Japanese insurance policies. FoundationOne®CDx Cancer Genomic Profile (F1CDx) (Cambridge, MA, USA) and the OncoGuide NCC™ oncopanel system (NOP) (Tokyo, Japan) are tissue-based CGP assays that were approved in December 2019. F1CDx examines 324 cancer genes, and reports known and likely pathogenic short variants, copy number alterations, and selected rearrangements [10, 11], while NOP examines 124 cancer genes, and also reports germline findings [12]. FoundationOne®Liquid CDx Cancer Genomic Profile (F1LCDx) is a liquid-based CGP assay that was approved in March 2021, with the same reportable genomic alterations as F1CDx [13]; F1LCDx was ordered only when appropriate tissue specimens were unavailable.

We referred to the OncoKB database for the definition of druggable genomic alterations [14]. Therapeutic evidence levels are classified into six levels in the database: evidence level 1 indicates genomic alterations with U.S. Food and Drug Administration-approved drugs. In this study, genomic alterations positioned as evidence level 1 in at least one cancer type, as well as microsatellite instability-high (MSI-H) or tumor mutational burden-high (TMB-H), were defined as druggable genomic alterations (Table 1) [15]. Available clinical trials included both investigator- and sponsor-initiated trials.

**Table 1:** Druggable genomic alterations

Gene symbol	Gene name		Alterations detail
*ATM*	ATM serine/threonine kinase	Oncogenic Mutations	
*ALK*	Anaplastic lymphoma receptor tyrosine kinase	Fusion	
*BRAF*	B-Raf proto-oncogene, serine/threonine kinase	V600E	
*BRCA1*	BRCA1, DNA repair associated	Oncogenic Mutations	
*BRCA2*	BRCA2, DNA repair associated	Oncogenic Mutations	
*BRIP1*	BRCA1 interacting protein C-terminal helicase 1	Oncogenic Mutations	
*CDK12*	Cyclin dependent kinase 12	Oncogenic Mutations	
*CHEK1*	Checkpoint kinase 1	Oncogenic Mutations	
*CHEK2*	Checkpoint kinase 2	Oncogenic Mutations	
*EGFR*	Epidermal growth factor receptor	Exon19 in-frame delition, Exon20 in-frame insertions, G719, L858R, L861Q, S768I, T790M	
*ERBB2*	Erb-b2 receptor tyrosine kinase 2	Oncogenic Mutations, amplification	
*FGFR2*	Fibroblast growth factor receptor 2	Fusion	
*FGFR3*	Fibroblast growth factor receptor 3	G370C, R248C, S249C, Y373C, fusion	
*IDH1*	Isocitrate dehydrogenase [NADP( +)] 1, cytosolic	R132	
*KRAS*	KRAS proto-oncogene, GTPase	G12C	
*KIT*	KIT proto-oncogene receptor tyrosine kinase	Oncogenic Mutations	
*NTRK1*	Neurotrophic receptor tyrosine kinase 1	Fusion	
*PALB2*	Partner and localizer of BRCA2	Oncogenic Mutations	
*PIK3CA*	Phosphatidylinositol-4,5-bisphosphate 3-kinase catalytic subunit alpha	C420, E542K, E545A, E545D, E545G, E545K, H1047L, H1047R, H1047Y, Q546E, Q546R	
*RET*	Ret proto-oncogene	Fusion	
MSI/TMB	Microsatellite instability/tumor mutational burden	MSI-H/TMB-H	

## Results

### Patient background

The tumors of 653 patients with 30 different types of cancers were analyzed with one of the three types of CGP assays. Of these, 571 patients (87.4%) were analyzed with the tissue-based CGP assay, and the most common cancer type was colorectal cancer (21.0%). In liquid-based CGP, pancreatic cancer was the most frequent type (40.2%) (Supplementary Fig. 1). Druggable genomic alterations were detected in 240 patients (36.8%). Detailed patient background information and druggable genomic alterations across cancer types are shown in Supplementary Table 1 and Supplementary Fig. 2, respectively.

### Genomic alteration types and availability of genome-matched therapies

**Fig. 1:**
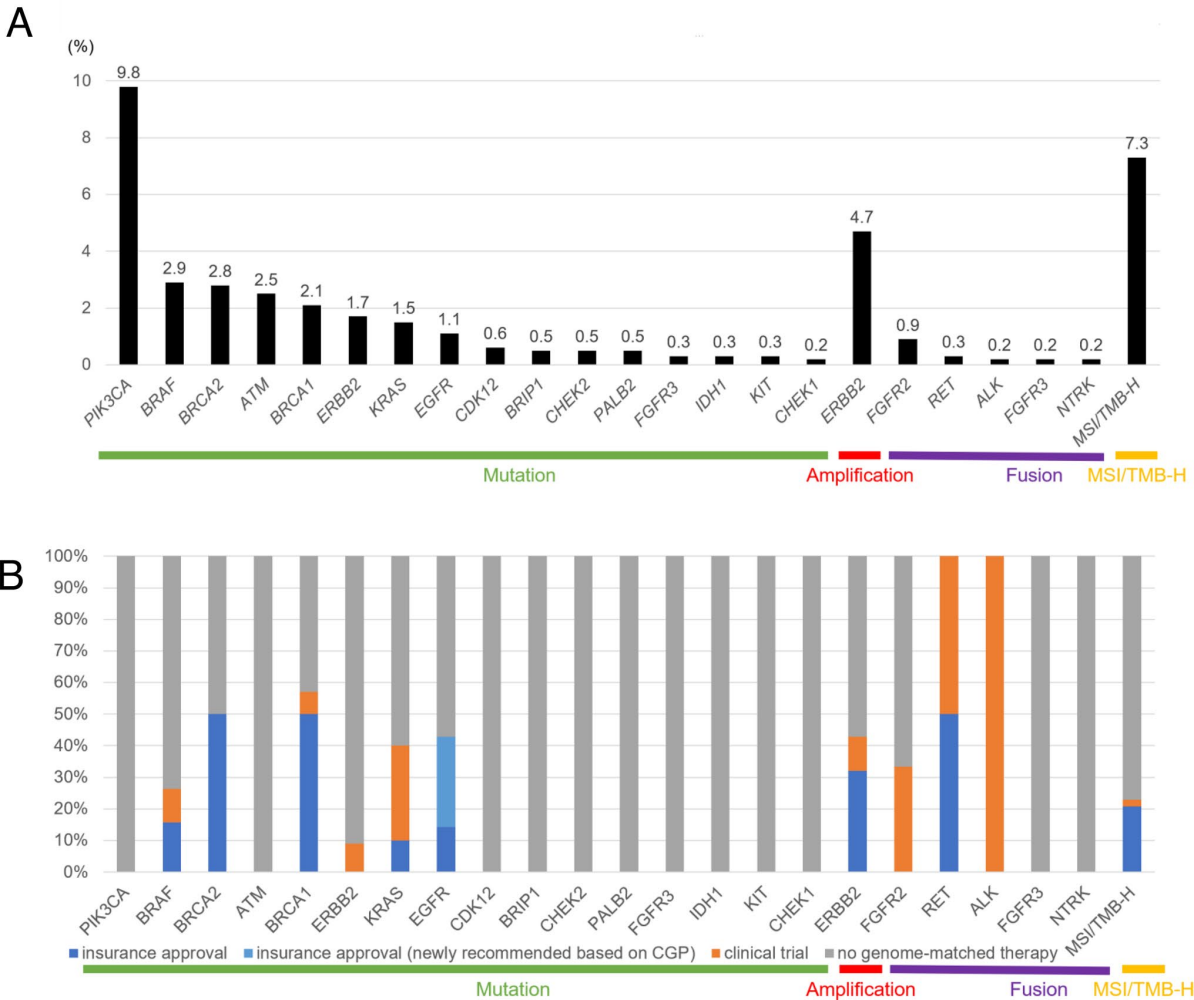
Landscape of druggable genomic alterations across gene types. **A** Detection rate. **B** Availability rate

*PIK3CA* mutations were the most frequent genomic alteration (9.8%), followed by MSI/TMB-H (7.3%) (Fig. 1A). The availability rate of pembrolizumab based on CDx or CGP results was 22.9% (Fig. 1B).

### Cancer type and availability of genome-matched therapies

**Fig. 2:**
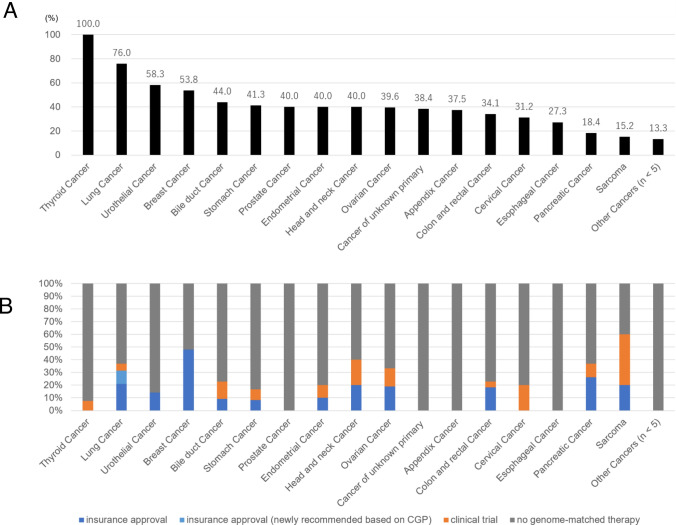
Landscape of druggable genomic alterations by cancer type. **A** Detection rate. **B** Availability rate

Sixty-two patients (9.5%) received genome-matched therapies, of which approved drugs and investigational agents accounted for two-thirds and one-third of the cases, respectively. The detection rate of druggable genomic alterations across cancer types is shown in Fig. 2A. While patients with thyroid and lung cancer harbored these alterations at high rates, those with pancreatic cancer and sarcoma had low rates of alterations (100%, 76%, 18.4%, and 15.2%, respectively). In contrast to the detection rate, the availability rate for genome-matched therapies was the highest among patients with sarcoma (60%) (Fig. 2B), followed by those with breast cancer, head and neck cancer (HNC), lung cancer, and pancreatic cancer (48%, 40%, 36.8%, and 36.8%, respectively). Of these, in terms of investigational drugs, the availability rates were high in patients with sarcoma and HNC (60% and 20%, respectively). Notably, in two patients (8.0%) with lung cancer, *EGFR* mutations (p.E709_T710delinsD and p.L861R) were newly detected, and they received osimertinib, an *EGFR* tyrosine kinase inhibitor (TKI). These variants were not detected by real-time polymerase chain reaction (RT-PCR) used as CDx for *EGFR* mutations.

### Barriers to accessing genome-matched therapies

**Fig. 3:**
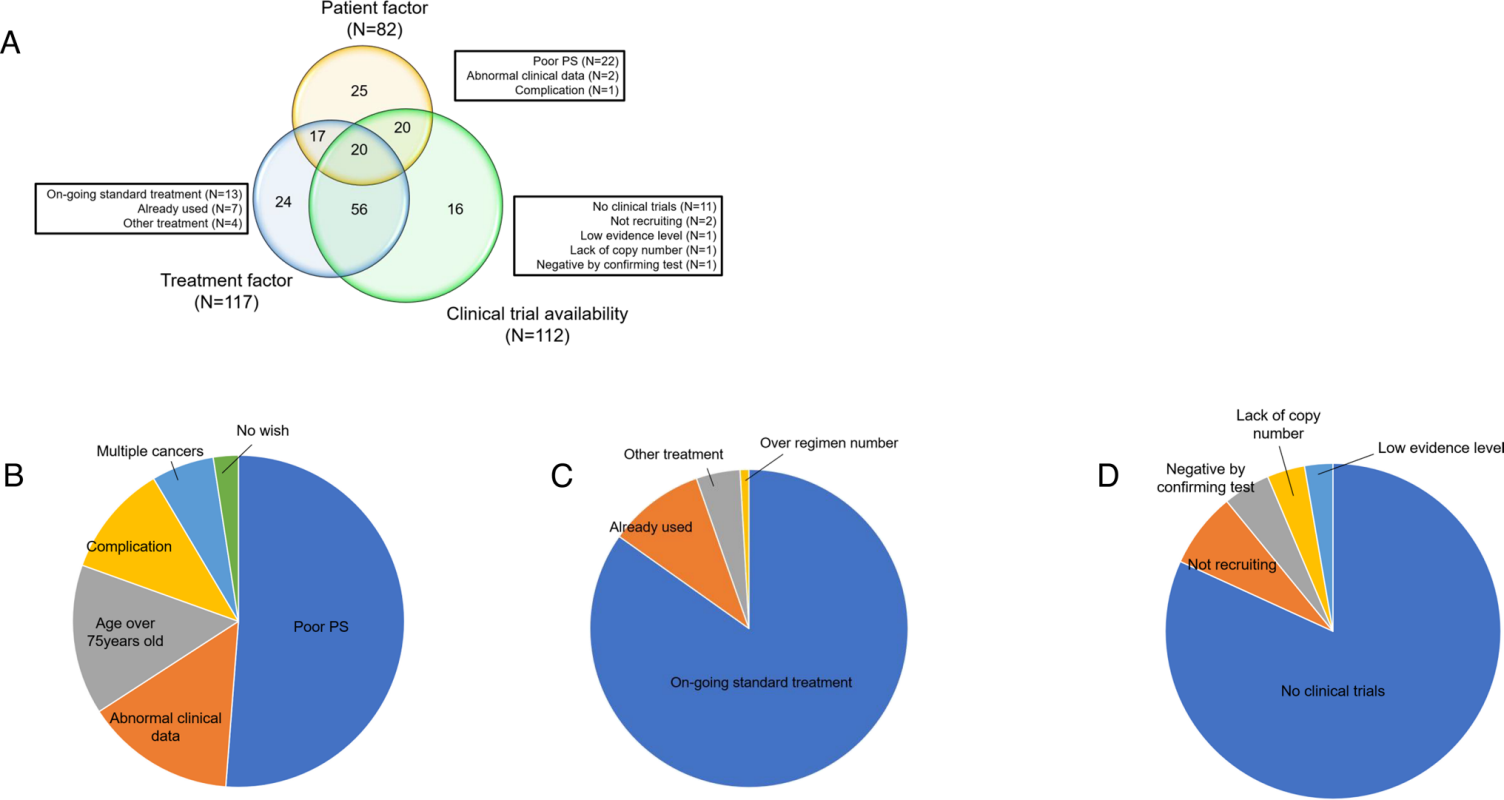
Multifaceted barriers in accessing genome-matched therapies and adaptations. **A** Overlap of each barrier. **B** Patient factors. **C** Treatment factors. **D** Clinical trial availability

In this study, 178 patients (27.3%) had druggable genomic alterations that were not actually applicable to any genome-matched therapies. Among these patients, we investigated barriers to accessing genome-matched therapies at the time of CGP results being returned. The most common reason was no available clinical trial (53.9%) and ongoing standard treatment (50.6%). Poor performance status (PS) accounted for 23.6%. We classified the barriers into three categories (i) patient factors such as poor PS or complications; (ii) treatment factors such as ongoing standard treatment; and (iii) clinical trial availability such as no clinical trial option or not recruiting. Figure 3A shows the overlap of each barrier with the others using a Venn diagram, and 113 patients (63.8%) had multiple barriers. Of 178 patients, 22 patients (12.4%) had poor PS only as a barrier to accessing genome-matched therapy. The details of the barriers are shown in Fig. 3B, C and D.

## Discussion

To the best of our knowledge, this is the largest study to evaluate the usefulness of CGP based on Japanese clinical practice, and it showed that rare cancers such as sarcoma and HNC are recommendable cancer types for CGP in addition to lung cancer. These results differ from the ESMO guidelines [7], which evaluated its usefulness in common cancer types mainly. Sarcoma and HNC are rare cancers [16, 17], and some reports indicated the usefulness of CGP as a treatment strategy for patients with rare cancers [6, 18]. In this study, the availability rate of genome-matched therapies was highest (60%) in patients with sarcoma. Physicians may often be hesitant to perform CGP on patients with sarcoma because they only harbor *TP53*, *ATRX*, and *RB1* mutations, and rarely harbor druggable genomic alterations [19]. However, this study demonstrated the importance of undertaking CGP even in patients with sarcoma. Sarcoma can be one of the phenotypes for hereditary cancer syndrome [20]. Performing CGP may be especially meaningful in younger patients because of its potential to detect secondary findings. The availability rate of genome-matched therapies was also high in patients with HNC (40%). There is significant heterogeneity in tumors derived from the head and neck region [21]. Of these, salivary gland carcinomas are genomically different from other HNCs because they have druggable genomic alterations such as *HER2*, *RET*, and *NTRK* [22]. In this study, two of the four patients who received genome-matched therapy were diagnosed with salivary gland cancer, which may have affected the results. However, CGP is actively recommended for patients with salivary gland cancer. Unlike sarcoma and HNC, the use of CDx before first-line treatment is well established in lung cancer, including adenocarcinoma histology [23]. This study demonstrated the usefulness of CGP even after CDx in these patients. Similar to a previous report, there were some patients (8% of total lung cancer patients) with *EGFR* mutations that could not be detected by RT-PCR [24].

Compared with other countries, the availability rate of genome-matched therapies other than approved drugs is still low. There are several possible reasons for this [25]. First, there are limited number of available clinical trials, as well as hospitals at which clinical trials are conducted. Moreover, these hospitals are concentrated in large cities in Japan, and distance is one of the barriers to participation in clinical trials for patients at local hospitals. Second, the Japanese universal healthcare system has rigid restrictions, and only patients who have finished standard treatment can undergo CGP. This system makes it difficult for patients to participate in clinical trials at the appropriate time. As our results demonstrated, most patients had multiple barriers to accessing genome-matched therapies, and 12.4% of patients could not be considered for these therapies solely because of the deterioration of their PS. For these patients, CGP at the front line could provide the opportunity to receive genome-matched therapies. In addition, several studies have reported genome-matched therapy can improved outcomes [8, 26]. Third, the compassionate use system is immature in Japan [27]. The establishment of more convenient patient access options such as a single-patient expanded-access clinical trial may enable genome-matched therapies and lead to better outcomes. Unexpectedly, the availability of pembrolizumab was low. This may be because TMB was approved as a CDx for pembrolizumab from March 2022. Notably, the availability of pembrolizumab was significantly different between the pre-approval and post-approval eras (17.9% vs 55.6%,* p* = 0.03). It was also considered important to provide physicians with feedback on the latest information as appropriate.

This study has several limitations. First, it is a single-institution retrospective study, and larger or prospective investigations are warranted to draw a definite conclusion about the status of CGP. Second, tissue-based and liquid-based CGP assays were not considered separately. TAT or the detection rate of druggable genomic alterations may differ between tissue and liquid-based CGP.

This study demonstrated the usefulness of CGP for patients with sarcoma and HNC in addition to lung cancer in clinical practice. Performing CGP at the front line has the potential to improve the availability of genome-matched therapy.

### Supplementary Information

Below is the link to the electronic supplementary material.

Supplementary file1 (PDF 78 KB)

Supplementary file2 (PDF 71 KB)

## Data Availability

The datasets generated during and/or analyzed during the current study are available from the corresponding author on reasonable request.
