# Supplementary figures and images for: Availability of genome-matched therapy based on clinical practice
Source: Int J Clin Oncol. 2024 Apr 26;29(7):964–71. doi: 10.1007/s10147-024-02533-z (PMC11196305; doi:10.1007/s10147-024-02533-z)

Supplemental Figure 1

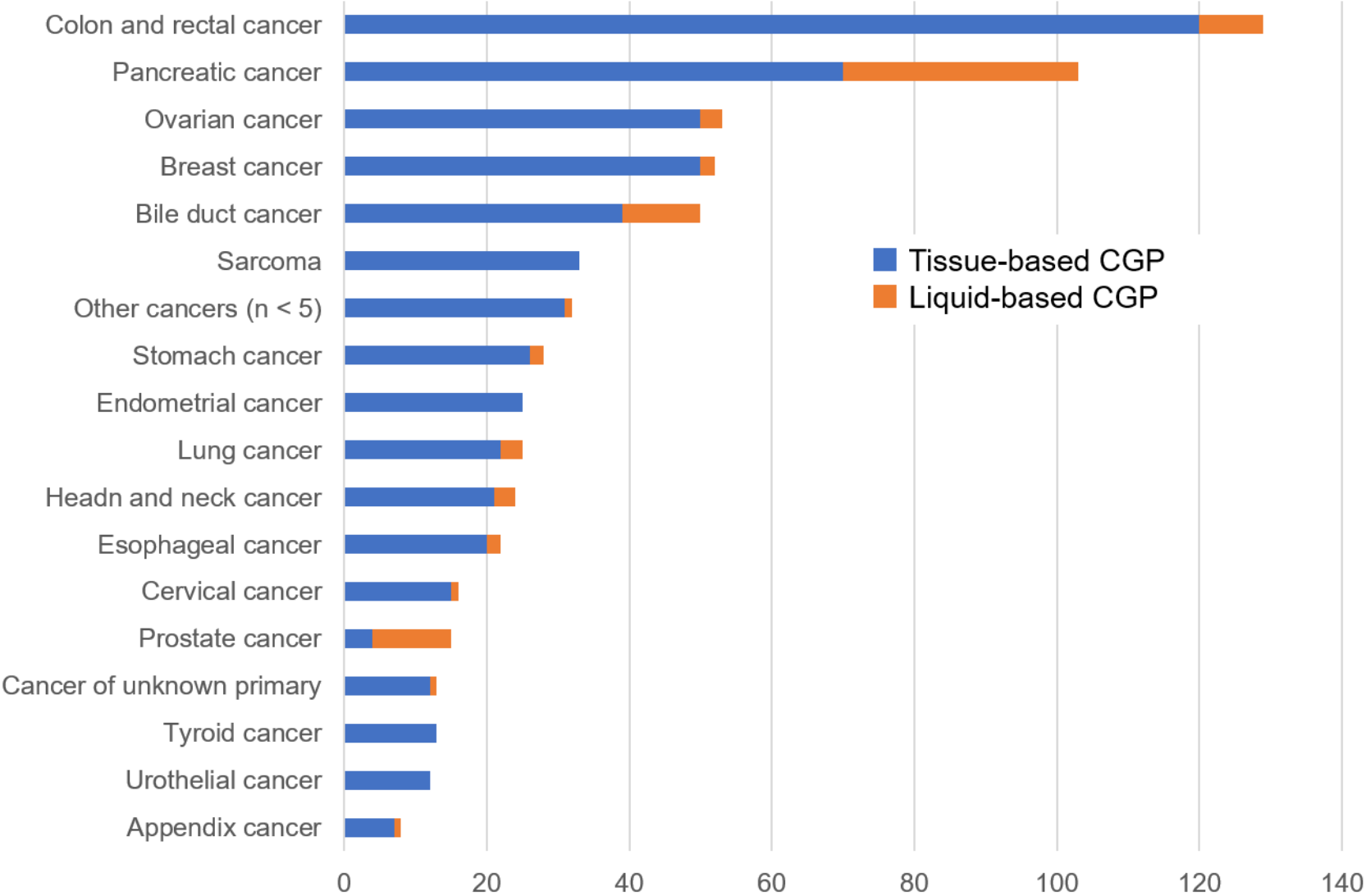

Supplement: Supplementary file 1 — Supplementary file1 (PDF 78 KB) [file 10147_2024_2533_MOESM1_ESM.pdf]

# Supplemental Figure 2

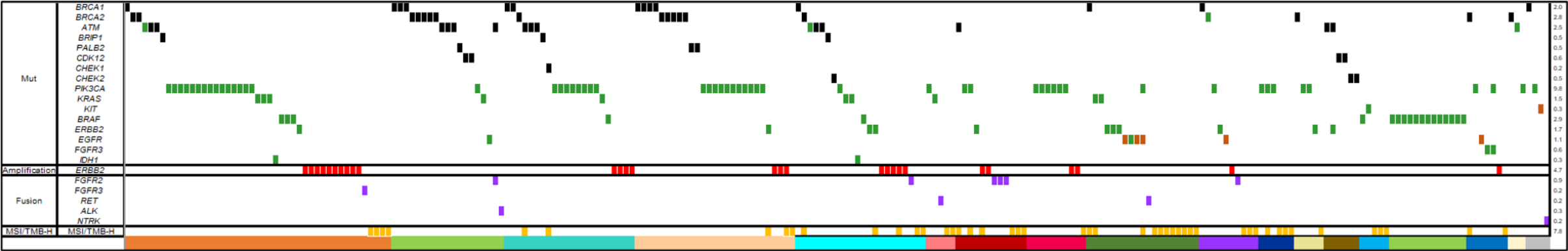

Supplement: Supplementary file 2 — Supplementary file2 (PDF 71 KB) [file 10147_2024_2533_MOESM2_ESM.pdf]
